# The Encapsulation of Anthocyanins from Berry-Type Fruits. Trends in Foods

**DOI:** 10.3390/molecules20045875

**Published:** 2015-04-03

**Authors:** Paz Robert, Carolina Fredes

**Affiliations:** Departamento de Ciencia de los Alimentos y Tecnología Química, Facultad de Ciencias Químicas y Farmacéuticas, Universidad de Chile, Santos Dumont 964, Independencia, Santiago 8380494, Chile; E-Mail: carolina.fredes@postqyf.uchile.cl

**Keywords:** food colourants, healthy foods, microencapsulation, spray drying, yoghurt

## Abstract

During the last decade, many berry-type fruits have been recognised as good sources of anthocyanins. Nevertheless, the use of anthocyanins in the development of food colourants and healthy and/or functional ingredients has been limited because of their low stability under given environmental conditions and interaction with other compounds in the food matrix. This review compiles information about the encapsulation of anthocyanins from twelve different berry-type fruit species as a technology for improving the stability and/or bioavailability of anthocyanins. Encapsulation by spray drying has been the primary method used to encapsulate anthocyanins, and some studies attempt to keep anthocyanin microparticles stable during storage. Nevertheless, more studies are needed to determine the stability of anthocyanin microparticles in food matrices over the product shelf life in the development of food colourants. Studies about encapsulated anthocyanins in simulated gastrointestinal models have primarily been conducted on the release of anthocyanins from microparticles to evaluate their bioavailability. However, adding anthocyanin microparticles to a food vehicle must guarantee the health properties attributed to the specific anthocyanins present in berry-type fruits.

## 1. Introduction

Berry-type fruits have long been regarded as having considerable health benefits because of their nutritional attributes, particularly their total antioxidant activity against cellular oxidation reactions [1]. These benefits have stimulated research to investigate the phenolic status and antioxidant activity of distinct berry fruit species and new varieties in different countries. There is a great variety of species from diverse botanical families (e.g., blueberries (*Vaccinium corymbosum* L., Ericaceae), strawberries (*Fragaria ananassa* Duch., Rosaceae), red raspberries (*Rubus idaeus* L., Rosaceae), and blackberries (*Rubus* sp., Rosaceae)) that produce the small purple or red fruits that are denoted as berries. In botanical terms, a berry is a fruit with many seeds and mesocarp flesh that evolves from a flower with a superior ovary [2]. Therefore, in strictly botanical terms, none of the above mentioned fruits are true berries. Nevertheless, these fruit species share a red-blue colour and a high polyphenol content and antioxidant activity. Because of the various botanical families and fruits to which berry-type fruits belong, a wide range of phenolic contents and corresponding antioxidant activities can be expected according to the specific compounds that are present in these species [3,4].

In recent years, other berry-type fruits from worldwide: bilberries (*Vaccinium myrtillus* L., Ericaceae), blackcurrant (*Ribes nigrum* L., Grossulariaceae), pomegranate (*Punica granatum* Linn., Punicaceae) and açaí (*Euterpe oleracea* Mart, Arecaceae), have also gained increased interest mainly due to the potential health benefits and the consumer demand of novel fruits [1].

Anthocyanins are responsible for the colour of berry-type fruits [5,6,7,8,9,10]. In addition to their colourant properties, anthocyanins have been associated with a wide range of biological, pharmacological, anti-inflammatory, antioxidant, and chemoprotective properties [11]. Anthocyanins are also beneficial against many chronic diseases [12,13]. New evidence highlights the potential use of berry-type fruit species as a source of bioactive compounds, primarily anthocyanins, in food and nutraceutical industries. However, the use of anthocyanin-rich extracts as food colourants and healthy foods is limited because of the low stability of anthocyanins under the environmental conditions (heat, oxygen, and light among others) experienced during processing and/or storage [14]. Anthocyanins are water-soluble pigments that correspond to the glycoside or acyl-glycoside of anthocyanidins and are stored in the plant cell vacuole [15,16]. The stability of anthocyanins is affected by pH, temperature, the presence of light, metal ions, oxygen enzymes, ascorbic acid, sugars and their degradation products, proteins and sulphur dioxide [17]. Additionally, anthocyanin bioavailability is low because of its sensitivity to pH changes. In general, anthocyanins are generally stable at pH values of 3.5 and below and they degrade at higher pH values [11]. Because the high instability of isolated anthocyanins from berry-type fruits has a direct impact on their colour stability and potential health benefits, encapsulation technology can be used to improve the stability and/or bioavailability of anthocyanins [18]. The objectives of this paper are to review the evidence regarding the encapsulation of anthocyanins from berry-type fruits by spray drying and to compile the new applications of anthocyanin microparticles in foods to propose future perspectives.

## 2. The Encapsulation of Anthocyanins from Berry-Type Fruits

Encapsulation is a technique by which active solid, liquid or gas compounds are introduced into a matrix or a polymeric wall system to protect the “actives” from environmental conditions, their interactions with other food components or to control their release (for a specific place and/or time) [19]. The polymers used in microencapsulation are called encapsulating agents (EA). The resulting microparticles are vesicles or small particles in which the size can vary from sub-microns to several millimetres. 

During the last four years, several studies on the encapsulation of anthocyanins from different berry-type fruits have been reported (Table 1). In all of these, anthocyanin encapsulation has primarily been focused on providing protection from environmental conditions (light, oxygen, temperature and water), avoiding oxidation and increasing the shelf life of active compounds. Currently, anthocyanin encapsulation is focused on studying the anthocyanins released in simulated gastrointestinal tracts [20,21,22,23,24,25]. 

**Figure 1:**
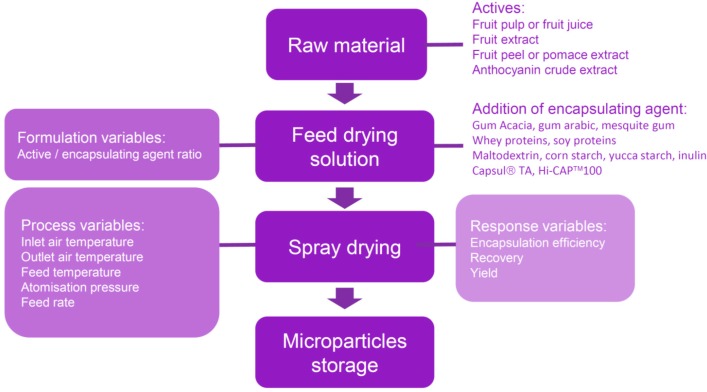
Microencapsulation of anthocyanins from berry-type fruits by spray drying and variables that must be considered in the feed formulation and process.

In the encapsulation of anthocyanins, fruit pulps, juices and extracts are selected from commonly consumed fruit species or exotic ones, and Andes berry (*Rubus glaucus* Benth., Rosaceae), bayberry (*Myrica* gole L., Myricaceae), black mulberry (*Morus nigra*, Moraceae), corozo (*Bactris guineensis*, Arecaceae), jaboticaba (*Myrciaria jaboticaba* (Vell.) O. Berg, Myrtaceae) and *kokum* (*Garcinia indica* Choisy, Guttiferae) have been used as raw materials. Pomace extract from bilberry [23,26], blackcurrant [27] and blueberry [24,25] and peel extract from jaboticaba [28,29] have also been used because anthocyanins are accumulated primarily in the fruit epicarp (peel) [30]. The anthocyanins from berry-type fruit have been encapsulated primarily by the oil dispersed phase, double emulsion (w/o/w), extrusion, emulsification/heat gelation, microgel synthesis, freeze drying, supercritical CO2, spray drying and ionic gelification (Table 1), but the most common method used to encapsulate anthocyanins from berry-type fruits is spray drying (Figure 1). Compared to the other encapsulation methods, spray drying allows one to obtain berry-type microparticle powders in a one step process. In addition, spray dryers are equipment commonly available in food and pharmaceutical industries.

### 2.1. Raw Materials

Several methods for treating fruits as raw materials for anthocyanin encapsulation have been described. The simplest method is to employ fruit pulp [31] or fruit juice [32,33,34,35,36] without any additional solvent; a filtration process is followed to eliminate solids before the preparation of a feed drying solution for the encapsulation process. The feed drying solution is obtained by homogenising the pulp or juice with one or more EAs. More steps are involved when either fruit pomace (PO) or peel (PE) are used as raw materials. Bilberry PO [23,37] commonly comes from pomace extraction with methanol, following filtration, evaporation and lyophilisation processes performed by providers. Bilberry PO also contains other polyphenols, tannins, carbohydrates and roughage [23,37]. Jaboticaba PE [28] comes from a peel extraction with ethanol (70%) that is acidified (pH 2.0) with hydrochloric acid, following filtration and evaporation processes. The feed drying solution is obtained in a similar way as that of the fruit extracts described above. 

**Table 1:** The encapsulation of anthocyanins from different berry-type fruits.

Raw Material	Encapsulation Method	Encapsulating Agent	References
Andes berry fruit extract	Spray drying	maltodextrin, gum Arabic, corn starch, yucca starch, Capsul^®^ TA (Ingredion Incorporated, Westchester, IL, USA), Hi-CA^TM^100 (Ingredion Incorporated, Westchester, IL, USA)	[38]
Bayberry fruit extract	Oil dispersed phase—spray drying	ethyl cellulose	[20]
Bayberry fruit juice	Spray drying	maltodextrin	[33]
Bayberry fruit juice	Spray drying	whey protein isolate or maltodextrin	[34]
Bilberry fruit extract	Emulsion	whey protein isolate	[21]
Bilberry fruit extract	Double emulsion (w/o/w)	pectin (calcium chloride), PGPR	[22]
Bilberry pomace extract	(a) extrusion; (b) emulsification/heat gelation; (c) spray drying	(a) amidated pectin; (b) whey protein isolate; (c) maltodextrin + pectin	[37]
Bilberry pomace extract	(a) emulsification/heat gelation; (b) extrusion	(a) whey protein isolate; (b) amidated pectin	[26]
Bilberry pomace extract	(a) extrusion; (b) emulsification/heat gelation; (c) spray drying	(a) amidated pectin; (b) whey protein isolate; (c) maltodextrin + pectin	[23]
Blackcurrant pomace	Spray drying	maltodextrin or inulin	[27]
Blackberry pulp	Spray drying	maltrodextrin	[39]
Blackmulberry fruit juice	Spray drying	maltrodextrin or gum Arabic	[35]
Blueberry fruit/pomace extract	Spray drying	whey protein isolate or gum Arabic	[24]
Blueberry fruit extract	Spray drying	mesquite gum	[ 40]
Blueberry pomace extract	Microgel synthesis	oxidized potato starch + sodium trimetaphosphate (STMP)	[25]
Corozo fruit extract	Spray drying	maltodextrin	[41]
Grape fruit juice	Freeze drying	maltodextrin + gum Arabic	[42]
Jaboticaba peel extract	Spray drying	maltodextrin, gum Arabic + maltodextrin or Capsul^TM^ + maltodextrin	[28]
Jaboticaba peel extract	(a) Rapid Extraction of Supercritical Solution (RESS); (b) ionic gelification	(a) polyethyleneglycol (PEG); (b) Ca-alginate	[29]
*Kokum* fruit extract	Spray drying	maltodextrin, gum Acacia or tricalcium phosphate	[36]
Pomegranate fruit juice or fruit extract	Spray drying	maltodextrin or soybean protein isolate	[32]

Some studies on anthocyanin encapsulation have described different methods of anthocyanin extraction from fruits before the preparation of the feed drying solution that involved more steps in the encapsulation process. Zheng *et al.* [20] have described the use of a microwave-assisted extraction method for bayberry fruits by using ethanol (80%) as a solvent and a rotary evaporation at 55 °C to remove excess solvent and some other low boiling point impurities. Similarly, other authors described the use of ethanol/water (1:1) for pomegranate arils [32], ethanol (96%) for blueberry fruits [40] and ethanol (80%) and citric acid (0.5%) for blackcurrant pomace [27]. Amberlite XAD16N column has been used to follow a solid-liquid fractionation of anthocyanins from whole blueberries and blueberry pomace with three different solvent systems (acetonic, ethanolic and methanolic) [24] and from corozo fruits without seeds with methanol/acetic acid [41], then the resulting extracts were freeze-dryed. 

### 2.2. Encapsulating Agents (EA)

A variety of EAs have been studied for anthocyanin encapsulation. Natural gums such as gum arabic [24,28,35,38], mesquite gum [40] and gum Acacia [36], proteins such as whey proteins [24,34] and soy proteins [32], polysaccharides such as maltodextrins of different dextrose equivalents [23,27,28,32,33,34,35,36,37,38,39], inulin [27], corn starch [38] and yucca starch [38], and modified polysaccharides such as Capsul^®^ TA [28,38] and Hi-CAP^TM^100 [38] have been successfully used in spray-drying. The choice of EA is very important for the proper encapsulation efficiency (EE), the stability of the active compounds in the microparticles during storage and the release properties in foods and the gastrointestinal tract. In the microencapsulation of anthocyanins, maltodextrin has been shown to be essential for preserving the integrity of anthocyanins for encapsulation [28]. Nevertheless, the use of this EA is limited by its solubility in water, and the subsequent release of anthocyanins in liquid media, which does not allow colour stability when the microparticles are applied in liquid foods such as dairy products [32].

### 2.3. The Encapsulation of Anthocyanins from Berry-Type Fruits by Spray Drying

Spray drying is widely used in the food industry to encapsulate active compounds and protect materials in an economic, simple and continuous way [19]. However, it is considered to be an immobilisation technology rather than a true encapsulation technology because some active compounds may be exposed superficially on the microparticles [43].

By using this technique, the feed or dispersion solution is sprayed (with a nozzle or a rotating disc), in the form of fine drops in a hot air flow. When the liquid droplets come into contact with the hot air, a resulting powder is instantaneously produced by the rapid evaporation of water [44]. In addition to the simplicity of spray drying, another advantage of this technique is that it is useful for encapsulating heat sensitive materials because the time of exposure to elevated temperatures is very short (5–30 s) [45]. Using this method, it is possible to obtain powder microparticles with low water activity that facilitate the transportation, handling, and storage of the product and ensure microbiological quality [44].

It is known that optimum drying conditions should be used to obtain a high encapsulation efficiency, which leads to the use of experimental design. The feed temperature, inlet air temperature and air outlet temperature (process variables) and active/encapsulating agent ratio (formulation variable) [44] have been reported as important variables in encapsulation efficiency, recovery, yield, and antioxidant activity (Figure 1). Inlet air temperature has been associated with anthocyanin oxidation and/or degradation reactions induced by heat. Different encapsulating agents have different optimum parameters because encapsulating agent features, such as solubility and viscosity, affect the formation rate of a crust on the particle surface [44,46]. The response surface methodology is applied to optimise the response variables that fit to some regression model, generally a second-order regression. For multiple optimisations, a desirable function is used.

A laboratory spray dryer (BÜCHI Labortechnik AG, Postfach, Switzerland) is generally used for anthocyanin encapsulation experiments and, in this instrument, the inlet air temperature can be regulated but the outlet air temperature varies according to the inlet temperature.

### 2.4. The Characterisation of Berry-Type Fruit Microparticles

The EEs represent the anthocyanin-polymer interaction from electrostatic interactions, or hydrogen bonding. Encapsulation efficiency is obtained by the quantification of the superficial and total anthocyanins [33]. Anthocyanin quantification is primarily achieved either colourimetrically or by high-performance liquid chromatography (HPLC). The principal method is the pH differential method by spectrophotometry, which is widely used in industry because it is a rapid and easy procedure to perform [47]. However, this method cannot provide any information regarding the compositional profile of anthocyanins and the relative amounts of these compounds. 

The EE of anthocyanins from berry-type fruits has commonly been determined by quantifying the total anthocyanins by spectrometry [22,23,24,25,28,29,32,33,36,39,40,42] or HPLC-DAD [21,23,26]. This profile indicates that the EEs of specific anthocyanins have not been reported in those studies. The EE of each anthocyanin may have special relevance when a fruit species has a wide profile of anthocyanins. For example, fifteen and thirteen anthocyanins have been identified in different bilberry [48] and blueberry [49] genotypes, respectively. Therefore, a wide range of EEs could be expected because of the different structural features of the anthocyanins identified in these fruits. However, there are not any studies on the structure-encapsulation efficiency relation. Additionally, the EE of specific anthocyanins can be crucial when the primary bioactivity and/or specific health effect of some fruits may be attributed to certain anthocyanins with specific structural features. Fang and Bhandari [33], using HPLC-DAD-ESIMS, have analysed the individual phenolics (gallic acid, cyanidin 3-glucoside, quercetin 3-galactoside, quercetin 3-glucoside and quercetin deoxyhexoside [tentatively identified]) of a bayberry juice solution before spray drying, the powders obtained immediately after spray drying, and the powders after 6 months of storage. The five phenolic compounds were found in the bayberry powders, with a high retention percentage (~93%–97%). Nevertheless, at the end of six months of storage, the phenolic contents declined at different rates, depending on the storage conditions. Cyanidin 3-glucoside was not detected when the powder was stored at 40 °C for 6 months, suggesting that the cyanidin 3-glucoside in bayberry powder is less stable than gallic acid and flavonols under the conditions of the study [33]. Different EAs could allow different EEs. Robert *et al.* [32] reported that the EE reached higher values for total anthocyanin than total polyphenol contents performed by spectrometry, showing the ability of maltodextrin MD (DE 12–20) and soybean protein isolate to bind anthocyanins. Thus, the flavylium cation could be related to the better polymer-anthocyanin interaction. This finding was consistent with Ersus and Yurdagel [50] who obtained the greatest pigment retention in the microencapsulation of anthocyanins from black carrot (*Daucus carota* L.) by spray drying with maltodextrin and DE 20–23.

The recovery from spray drying was reportedly influenced by the encapsulating agent properties (viscosity and solubility), anthocyanin/encapsulating agent ratio, and the inlet air temperature. A high recovery of anthocyanins could be attributed to short drying times or the rapid formation of a dry crust, which allows for water diffusion but retains the active properties. 

Some studies on the stability of anthocyanin microparticles during storage have been reported [27,32,33]. Pseudo-first order kinetics for encapsulated anthocyanins from pomegranate juice and pomegranate ethanolic extract with maltodextrin (DE 12-20) and soybean protein isolate have been reported [31]. The storage conditions of anthocyanin microparticles, such as the polymer nature, temperature, and water activity (aw), among others influence their stability. The effect of the polymer’s nature was observed in encapsulated blackcurrant with different maltodextrins (DE 11, DE 18 and DE 21) and inulin during storage at 8 °C and 25 °C [27]. Anthocyanins were significantly more stable in inulin, and no effect from the DE in maltodextrins was found [27]. In encapsulated pomegranate juice with maltodextrin (DE 12-20) and soybean protein isolate stored at 60 °C, maltodextrin showed the best anthocyanin protective effect, and it had the lowest degradation rate constant [32]. The effect of the aw was studied in encapsulated bayberry juice, with maltodextrin DE10 stored at different temperatures (4 °C, 25 °C and 40 °C) and aw amounts (0.11, 0.22, 0.33 and 0.44) [33]. At a higher aw during storage, the degradation of anthocyanins increases [33]. The storage temperature plays an important role in the stability of anthocyanins, which is associated with the glass transition temperature of a spray-dried powder (rubber-glassy transition).

Other studies have been conducted with spray-dried juice using carrier agents although the encapsulation parameters were not evaluated [31]. In this context, spray-dried açai juice with maltodextrin (DE10 and DE20), Arabic gum and tapioca starch with aw of 0.33 and 0.53 were stored at 25 °C and 35 °C. The maltodextrin DE10 and the lower aw powders showed the best anthocyanin protection [31].

## 3. Applications in Foods

### 3.1. Anthocyanins as Colourants

Stability is a crucial factor to consider when anthocyanin pigments are used as food colourants. This issue has special relevance when natural colourants (based on anthocyanins) are compared with synthetic ones. Arocas *et al.* [51] compared the colour stability of three natural red colourants (cochineal (E120), enocyanin (E163) and dark carrot (E163)) and three artificial colours (allura red (E129), Carmoisine (E122) and Ponceau 4R (E124)), determining major differences in the natural colours’ responses to changes in pH (3–8) and temperature (80 °C for 30 min), thus giving them a lower stability in relation to artificial colourants. Fracassetti *et al.* [52] evaluated the storage effects on the total and individual anthocyanin content of a lyophilised powder (used as a colourant) from wild blueberry (*Vaccinium angustifolium*) at 25 °C, 42 °C, 60 °C, and 80 °C for 49 days. The storage reduced the total and individual anthocyanin contents, and the reduction was slower at 25 °C (after 2 weeks), whereas it was more rapid at 60 °C and 80 °C after 3 days. Based on this result, the half-lives were determined to be 139, 39, and 12 days at 25 °C, 42 °C, and 60 °C, respectively. This result is less than five months at 25 °C. 

The interaction of anthocyanins with ascorbic acid (AA) is particularly noteworthy because the effect of this interaction is largely negative. West and Mauer [53] have studied the colour and chemical stability of six anthocyanins (highly purified and present in semi-purified extracts) from grape pomace, purple corn, and black rice, in combination with ascorbic acid solutions at different pH values (3.0 to 4.0) and temperatures (6–40 °C), and in lyophilised powders at different relative humidity values (43%–98% RH). The results indicated that in liquids, stability was negatively correlated with increased pH and temperature, and that for powders, stability was inversely proportional to the relative humidity, also confirming the mutual destruction of the anthocyanins and ascorbic acid in solution. This finding is particularly important for beverages because they form a product category in which anthocyanins and AA are part of the same formulation. 

The first antecedents of anthocyanin application in yoghurt were based on the addition of açai juice [54] and lyophilised fruit from *Berberis boliviana* [55] to commercial yoghurt. Nevertheless, these successful examples differ from the results of studies about anthocyanin addition during the process of yoghurt formulation. Karaaslan *et al.* [56] have evaluated the incorporation of wine grape ethanolic extracts before the yoghurt production process, noting a significant degradation of anthocyanins during storage. Similar results were reported by Sun-Waterhouse *et al.* [57], who indicated that fermentation affects the anthocyanin content of blackcurrant extract during the yoghurt formulation process. Additionally, Scibisz *et al.* [58] determined that certain probiotic cultures significantly affect the stability of anthocyanins from cranberry fruit when added to yoghurt.

Microencapsulation has been used as a strategy to address the stability of anthocyanins that are used as natural colourants. Robert *et al.* [32] compared the degradation kinetics of encapsulated and un-encapsulated anthocyanins from pomegranate juice, obtaining similar degradation constants when they are applied to yoghurt, showing the loss of the protection after addition to the yoghurt. This finding may be explained by the solubility of the EA in hydrophilic matrices. Therefore, the selection of an EA that allows microparticle dispersion may be an alternative to designing a dairy product such as yoghurt with natural colourant based on a berry-type fruit.

### 3.2. Anthocyanins in Healthy Foods

In recent years, encapsulation technology has increased in importance in the food industry, particularly in the development of functional and/or healthy foods. The use of encapsulated anthocyanins as an ingredient in healthy foods should allow for the protection of the anthocyanins (the preservation of their nutritional properties) until they are consumed within the food vehicle [43]. However, to the best of our knowledge there are not studies of anthocyanin release from microparticles in food matrices. To find the kinetic release of anthocyanins from release curves in food, the data are fitted to mathematical models (Peppas [59], Higuchi [60] and Hixson-Crowel [61]), allowing researchers to obtain the release rate constants and to explain the release mechanism. When the anthocyanin microparticles are soluble in the food matrix, the anthocyanins are quickly released. Without this protection, the anthocyanins would otherwise be exposed to adverse conditions in the food (pH, enzymes, and other food components). In this case, the microparticles would be best suited to functional dry-mixes or instant food. Contrary, when microparticles are insoluble in food matrices, the release is very slow and they could be used for the formulation of liquid functional-foods. Therefore, the solubility of encapsulating agent determines the applicability of the microparticles in foods.

Some studies have been conducted on the effect of process variables of the spray drying (inlet air temperature, outlet temperature, feeding rate, and polymer nature (EA)) on the antioxidant capacity (measured by ABTS or DPPH) of berry-type fruit powders [23,27,40]. In all of them, a reduction of anthocyanin content (mainly due to the inlet air (140–205 °C) and outlet temperature) led to a decrease in antioxidant capacity, the last being used as a response variable [23,27,40]. In other work, the effect of storage temperature and aw on the antioxidant capacity of bayberry powders was undertaken [33]. 

The information about EA was evaluated for anthocyanin controlled release in simulated gastrointestinal digestion models [20,21,22,23,24,25], and they indicated the use of ethyl cellulose [20], whey protein isolate [21,23,24], pectin, PGPR [22], amidated pectin [23], maltodextrin + pectin [23], gum arabic [24], and potato starch/STMP [25]. 

Kropat *et al.* [26] have determined the anthocyanin release in cell culture from two applied microencapsulation systems (Table 1). Fifteen different anthocyanins, composed of five aglycons called delphinidin (del), cyanidin (cy), petunidin (pt), malvidin (ma), and peonidin (peo) and three glycons called glucose (glc), galactose (gal), and arabinose (arab), were identified in bilberry extract by HPLC-DAD, where del-glycosides (34.3% wt) and cy-glycosides (27.6% wt) predominated. Although the anthocyanins were stabilised by microencapsulation, the rate of the decrease in the anthocyanin content appeared to be substantially affected by their respective aglycon. Del-glycosides disappeared completely within 30 min, whereas cy-, pt-, ma-, and peo-glycosides were relatively stable.

## 4. Future Perspectives

During recent years, the official rules of the European Union (EU) and the United States have restricted the use of synthetic colourants (especially red ones) as food additives because of their potential adverse health effects. These effects are related to their carcinogenic effects in experimental models [62,63], possible allergenic effects [64], and hyperactivity in children (3 and 8–9 years) [65]. McCann *et al.* [65] have evaluated the mixture of synthetic colourants with sodium benzoate (frequently used in soft drinks and other foods), considering among the synthetic red colourants azorubine red (Carmoisine (E-122), Ponceau 4R (E-124) and Allura red AC (E-129)). Their findings indicated that childhood hyperactivity could be exacerbated by the use of synthetic colourants. Since the establishment of this evidence, the European Food Safety Authority has decreased the allowed daily intake levels of these synthetic colourants. Additionally, Chilean Food Health Regulations do not allow the use of colourants in infant foods (until 3 years) [66]. Therefore, international regulation trends in addition to informed consumers are a market opportunity for natural food colourants based on anthocyanin pigments in which microencapsulation is a key technology for the development of natural colourants that are stable in food matrices.

Studies about encapsulated anthocyanins in simulated gastrointestinal models have primarily been conducted on the release of anthocyanins from microparticles without using a food vehicle [22,23,24,25]. The use of encapsulated anthocyanins as ingredients in healthy foods should allow for the protection of anthocyanins until reaching the gastrointestinal site where anthocyanin release is desired. 
